# Cost of operating a human milk bank in the UK: a microcosting analysis

**DOI:** 10.1136/archdischild-2024-327543

**Published:** 2024-09-26

**Authors:** Hema Mistry, Gillian Weaver, Natalie Shenker

**Affiliations:** 1 Warwick Clinical Trials Unit, University of Warwick, Coventry, UK; 2 The Human Milk Foundation, Berkhamsted, England, UK; 3 Department of Surgery and Cancer, Imperial College London, London, UK

**Keywords:** Health Care Economics and Organizations, Neonatology

## Abstract

**Objective:**

Globally, demand for donor human milk (DHM) is increasing with WHO guidelines recommending DHM as the first line nutrition for premature infants in the absence or shortfall of maternal milk. Policymakers and clinicians currently have limited knowledge regarding costs incurred by human milk banks (HMBs) making the planning and resourcing of these services challenging. This study aimed to evaluate costs in a national network of HMBs in the UK.

**Design and setting:**

All 14 UK HMBS were invited to complete a bottom-up microcosting survey from 1 April 2021 to 31 March 2022 covering four key areas: Staffing, equipment, donor screening and incidental costs. Total annual salary costs included on-costs (ie, national insurance, pensions), qualifications, overheads (ie, non-staff costs) and capital overheads. The annual equivalent costs for each equipment item were based on the total cost over its useful life and discounted at 3.5%.

**Results:**

10 out of 14 milk banks provided responses with more complete datasets returned by larger milk banks. Staffing costs ranged from £24 983 to £476 194 (n=9, mean: £159 798) and considerable expertise was provided voluntarily or from non-costed staffing. Other costs included equipment (n=7, range: £2600–£51 453, mean: £19 279), donor screening (n=6, range: £925–£38 057, mean: £18 570), incidentals (n=6, range: £650–£109 996, mean: £23 774). The total annual cost of operating a milk bank annually was £202 719 (range, £27 583–£675 699) to provide between 205 and 3495 litres of DHM. The cost per litre averaged £173.49 (range, £95.94–£274.88).

**Conclusions:**

The average cost of DHM is currently higher than current cost recovery tariffs and this study likely under-represents total costs. This study provides useful benchmarking data for future economic analyses, trial development and service planning.

**WHAT IS ALREADY KNOWN ON THIS TOPIC**

**WHAT THIS STUDY ADDS**

- Microcosting enables a detailed economic evaluation of all inputs into service delivery giving precise estimates of their true costs. This study is also the first to survey costs of milk banking across a single country in 10 milk banks demonstrating variability between the ability to report comprehensive budgets. The considerable variation in the reported cost of providing DHM across the 10 milk banks likely is explained by the under-reporting of costs, particularly by smaller milk banks that serve a single neonatal unit.

**HOW THIS STUDY MIGHT AFFECT RESEARCH, PRACTICE OR POLICY**

- The current study results are in line with previously reported studies and demonstrate that current tariffs for DHM used in hospitals may not be adequate to cover human milk bank costs. This evidence will be useful for planning and assessing the impacts of future clinical trials, planning future sustainable milk banking services in the UK and other high-resource settings as well as providing a set of tools that can be used for cost evaluations in low and middle-income settings.

## Introduction

 Although breastmilk is the optimal infant nutrition,^1^ not all mothers can provide sufficient milk to meet the needs of their infants. Recent global and UK national guidance recommends donor human milk (DHM) in the absence or shortfall of maternal milk for premature infants.^2 3^ DHM is defined as ‘breast milk expressed by a mother that is then processed by a milk bank for use by a recipient who is not the mother’s own baby’,^4^ and its use reduces the incidence and severity of complications of prematurity.^5^,^7^ As part of a lactation support programme, DHM can be supportive of maternal lactation and breastfeeding^8 9^ and support parental well-being.^10^,^12^ However, very few studies have reported the true costs of operating human milk banks (HMBs) meaning services risk being financially unsustainable.

Currently, there are 14 HMBs in the UK including a national service in Scotland, a single HMB in Northern Ireland that also supports neonatal units in the Ireland and 12 HMBs ranging in size from those that supply a single neonatal unit to regional HMBs that supply single or multiple operational development networks; while there is no HMB in Wales, neonatal units have access to DHM through a hub based in Swansea or from milk banks in England.^13 14^ The National Institute for Health and Care Excellence published a guideline in 2010 providing recommendations for HMB operations, covering recruitment, milk donor screening and processing.^4^ HMBs continue to vary in their management, size and output, with differing staffing, activity levels and screening.^15^ There is also significant variation between neonatal units in the criteria for DHM use with limited trial data.^16^,^18^

To the best of our knowledge, no study exists globally that provides the costs of DHM or the running costs of HMBs across a national network. Here, we report on the results of a UK costing survey where a bottom-up costing exercise explored variations in how different UK HMBs are run and to account for variations in the unit costs of providing DHM across the UK.

### Methods

The survey was carried out between October 2022 and May 2023 to assess costs between 1 April 2021 and 31 March 2022. A cost survey was developed between the co-authors using Microsoft Excel covering five key areas: Staffing, equipment, incidental, screening and other information.

- Staffing costs included the number of staff, either paid or unpaid (including volunteers), the number of full-time equivalent posts, the role of the staff members, the average pay band for each staff member along with their hourly wage rate and the number of hours they worked each week.
- Equipment costs (number and item cost), depreciation term (in years) and maintenance costs. The following items were included: Pasteurisers, freezers, fridges, dishwashers, washing machines, biosafety cabinets, blast chillers, computers/laptops, printers or any other equipment.
- Incidental costs included overheads, cleaning, couriers, contingencies, printing, postage or other consumables.
- Screening costs included the cost of thermometers, containers, serological and microbiological screening and other screening costs.
- Any other information included the number of donors recruited and the number of litres of milk pasteurised, discarded and issued overall.

#### Staffing costs

Annual salary costs for each staff member were based on Agenda for Change bands (table 1). The total annual salary costs also included any on costs (ie, national insurance, employer pension costs), qualifications, overheads (ie, non-staff costs) and capital overheads calculated as reported in the Personal Social Sciences Research Unit report on the unit costs of healthcare as actual numbers.^19^

**Table 1:** Unit costs cost for salary scales

Annual pay scales 2021/2022	Salary cost(midpoint/top point*)	Source
Band 2Band 3Band 4Band 5Band 6Band 7	£19 918*£21 777*£24 882*£27 780£34 172£42 121	https://www.nhsemployers.org/articles/annual-pay-scales-202122

Note: In addition to the full-time equivalent basic salary for the Agenda for Change bands for staff earnings, we also added in any costs relating to on-costs (ie, national insurance), qualifications, overheads (ie, non-staff costs) and capital overheads to generate the actual annual salary cost.^19^

#### Equipment costs

For some items of equipment, only the number of items was provided. If the brand was provided, we searched the internet using Google to obtain its price; if the name was not provided, we used an average cost from other HMBs. This approach was also used for the average lifespan (in years) of equipment. Maintenance costs were included if the HMB provided them or estimated if the HMB indicated there was an annual maintenance cost. The annual equivalent cost was then calculated for each individual equipment item based on the total cost over its useful life (ie, 5 or 10 years) and discounted at 3.5%, the rate recommended by HM Treasury.^20^

#### Incidental and screening costs

The costs were included for any incidental items and consumables such as printing, postage, containers, lids, jugs and trays among other items as well as any screening costs as recorded by individual HMBs.

### Statistical analysis

We used bottom-up analysis (microcosting) to provide a detailed analysis of the costs allowing for variation across different HMBs and to provide a cost per litre of DHM. All unit costs are presented in 2021/2022 prices and adopted a National Health Service (NHS) perspective.

## Results

10 out of the 14 HMBs provided a response to the costing survey with survey completeness indicated in table 2. Most HMBs provided information on staffing costs and the volume of milk that was pasteurised and issued. Information returned for the other categories was variable.

**Table 2:** Level of completed data from costing survey

Milk bank	Staffing costs	Equipment costs	Incidental Costs	Screening Costs	Other*	Milk volume
**A**	N	N	N	N	P	Y
**B**	Y	P	N	N	N	Y
**C**	Y	P	Y	N	N	Y
**D**	P	N	N	N	P	P
**E**	Y	Y	Y	Y	P	Y
**F**	Y	P	P	Y	N	Y
**G**	Y	P	P	Y	N	Y
**H**	Y	P	P	Y	N	Y
**I**	Y	N	N	N	N	N
**J**	Y	P	P	Y	N	Y

* The milk banks provided some information to help with costing.

N, unknown/not completed; P, partially completed; Y, fully complete.

### Staffing costs

Two of the 10 HMBs (A and D) did not provide complete information on staff roles. Eight HMBs had an HMB coordinator or manager with variable full-time equivalent (FTE). HMB I had two coordinators working 0.11 FTE (4 hours) and 0.2 FTE (7.5 hours) per week. HMBs G and J had a coordinator/manager who worked 1.0 FTE per week supported by various paid staff members including admin/logistics (n=5), lactation support (n=2) and technical (laboratory) support (n=5).

Many HMBs were also supported by unpaid staff including clinical consultants (n=7), microbiologists (n=7), neonatologists (n=5), paediatricians (n=3), IT support (n=6) and other staff members such as an operations director or infant feeding advisors (n=4). These staff members were paid from other contracts rather than the HMB budget. Four HMBs also reported the number of volunteers who assisted operations in their HMBs. None of these costs for the unpaid staff members and volunteers were included in this costing analysis.

Total staff costs averaged £159 798 (n=9) and ranged from £24 983 (HMB B) to £476 194 (HMB E).

### Equipment costs

Seven out of the 10 HMBs provided information on equipment costs. Only HMB E comprehensively completed this information; 6 HMBs partially completed this equipment. HMB H reported the number of equipment items but not their costs. The most expensive items were pasteurisers costing between £25 000 and £38 000; 5 of the 7 HMBs providing costs had two pasteurisers. The next significant equipment costs for the HMBs were biosafety cabinets (range £7,000–£8,000) and nutritional analyser costing approximately £25 000 (plus up to £5000 annual operating costs depending on use). Only one HMB (HMB E) provided information on the average lifespan of the equipment which allowed assumptions to be made for the equipment lifespan of the other HMBs. Maintenance schedules were not reported by some HMBs, hence these HMBs were undercosted.

Total costs averaged £19 279 (n=7) and ranged from £2600 (HMB B) to £51 453 (HMB E). The low cost for HMB B reflects only the tracking and tracing system cost was reported.

### Screening costs

Six out of the 10 HMBs provided information on recruitment and screening costs including costs for DHM containers and donor freezer thermometers; 4 HMBs provided costs for microbiological screening.

The mean screening cost was £18 570 (n=6) ranging from £925 (HMB C which provided only costs for donor containers and thank you notes) to £38 057 (HMB E who provided a comprehensive breakdown of costs). Not all HMBs pay for microbiology costs as these are absorbed within their NHS Trust pathology department budget for their NHS Trust.

### Incidental costs

Six out of the 10 HMBs provided information on incidental costs. Two of the Trusts (HMBs C and G) explicitly reported that their incidental costs are covered by their Trusts. Incidental costs averaged £23 774 (n=6), ranging from £650 (HMB H) to £109 996 (HMB E).

### Overall costs and the cost per litre of DHM

Table 3 provides the total annual cost of running the HMB which ranged from £27 583 (HMB B) who provided only staffing costs and some equipment costs to £675 699 (HMB E) who provided a comprehensive breakdown of all costs. The mean total costs were £202 719 (n=10).

**Table 3:** Cost per litre of donor human milk

Milk bank	Total cost*	Total litres of usable milk issued per year	Cost per litre
**A**	£100 000	725	£137.93
**B**	£27 583	288	£95.94
**C**	£52 721	205	£257.17
**D**	£200 000	1560	£128.21
**E**	£675 699	3495	£193.33
**F**	£164 930	600	£274.88
**G**	£165 799	1286	£128.93
**H**	£294 744	2090	£141.03
**I**	£83 411	Unknown	†
**J**	£262 306	1436	£182.66
**Average**	**£202 719**	1298	**£173.49**

* This total cost is based on what was provided for by each milk bank which is undercosted as not all items for each of the four categories (staffing, equipment, incidental and screening) were recorded in the cost survey.

† Unable to estimate.

Nine of the 10 HMBs provided the total number of usable litres of milk issued annually which ranged from 205 litres (HMB C) to 3495 litres (HMB E); therefore, the cost per litre of milk ranged from £95.94 (HMB B) to £274.88 (HMB F). Across the 10 HMBs, the average cost per litre of milk was £173.49.

## Discussion

This paper reports a recent cost data collection template completed by 10 HMBs in the UK. To the best of our knowledge, this is the first national HMB infrastructure costing survey.

Of the 10 out of 14 HMBs that provided costing information, the average cost per litre of DHM ranged from £95.94 to £274.88 (average, £173.49). Staffing costs contributed the majority of the cost per litre. However, these results need to be interpreted with caution as some of the information provided was incomplete or highlighted variable funding sources. For example, one HMB is funded directly by NHS England for staffing, equipment and consumables while incidental costs such as cleaning and electricity are funded by the NHS Trust that hosts the HMB and were unreported. Another stated that the HMB costings were incorporated within the neonatal unit budget so a full costing breakdown was not possible. Moreover, some HMBs explicitly stated that due to the lack of staff time, they were unable to provide full costs.

While no association was found between the cost of DHM provision and volumes processed annually, this likely resulted from the under-reporting of costs by the smaller HMBs which are more likely to be embedded with a neonatal intensive care unit. Economies of scale are likely to be very relevant and some of the most expensive equipment (eg, pasteurisers, biosafety cabinets, nutritional analysers) are largely under-used in small and medium-sized banks with implications for service effectiveness and safety. As each pasteurisation run typically takes between 2–3 hours, the total amount of milk that could be processed each week if the pasteurisation equipment was used differently would increase markedly if more than one device was used in parallel decreasing the overall staff time and ensuring the maximum efficiency from the equipment and space.

The only previous published evidence from the UK of DHM costs came from a report examining breastfeeding promotion in UK neonatal units which calculated the cost per litre as £289.12 (£395.48 based on 2021/2022 prices; unpublished report for Department of Health, 2005).^21^ The average cost per litre has been reported in Italy as €231 (approximately £198, 2019 prices)^22^ and €306.95 in Germany (approximately £264, 2017 prices)^23^ but these reports only investigated a single HMB. A 2017 systematic review found that the cost of DHM could be as little as $8.04 (£6.30) per 100 mL in Sweden (US dollars in 2015 prices).^24^ The results from our costing survey are in line with these other studies with missing data likely contributing to an underestimation of the true costs.

Feeding infants with screened DHM in the absence or shortfall of maternal milk is significantly more expensive than using infant formula^23^ where the cost reported from the NHS Supply Chain in 2019 for 100 mL specialist preterm formula was £0.53.^25^ However, savings are created as a result of reducing necrotising enterocolitis (NEC) rates, reduced time to full feeds, shortened durations of hospital stay and other health impacts and a systematic review based on NEC reduction alone has highlighted significant potential cost-savings.^26^ Other studies have demonstrated that DHM availability may also be associated with improved parental well-being and infant health as well as promoting, protecting and supporting breastfeeding;^11 12^ as well as supporting donor well-being.^27^ The impact on healthcare costs and quality of life outcomes of these effects remain unexplored but need to be evaluated as part of future cost-effectiveness analyses. While the total cost of providing DHM is very likely underestimated in this study, the cost-effectiveness of milk banking services may also have been underestimated.

Understanding the costs of HMB services will be essential in supporting future planning for equitable services and to develop much-needed randomised controlled trials in populations beyond extremely premature infants. HMB funding currently varies across the UK with smaller HMBs largely funded directly through their neonatal unit budget^15^ while regional HMBs are funded directly from NHS England or through cost recovery from DHM provided to neonatal units; the Scottish National Milk Bank Service is funded by contributions from each regional health board according to a formula calculated from population data and deprivation index. At the time of the study, tariffs for a litre of DHM varied from £100–£150 per litre (about £5–£20 per infant per day)^15^ and have generally not increased since 2009.^21^ Although, the full cost implications of managing an NEC diagnosis have not been described in the UK, estimates extrapolated from studies in the USA suggest that the total cost on in-patient stay can range from US$225 000 to US$880 000 (approximately £175 000–£700 000) if peritoneal drainage and/or laparotomy is needed.^28^ Other costs of long-term at-home total parenteral nutrition, gut transplantation and economic impacts on the family as a whole need estimation but are likely to run into many £100 000s. As the use of DHM extends to other populations, full-cost evaluations need to be conducted to evidence the cost-benefit and cost-effectiveness of this intervention as part of a programme of lactation support.

Despite many HMBs receiving inadequate remuneration to cover costs,^29^ preserving safety and supporting the milk donor emotionally and practically is paramount.^30^ Future studies should evaluate the gold standard of care that HMBs should provide to milk donors and how much it costs to provide. DHM has recently been classified as a substance of human origin (SoHO) in the revised European Union Directive on Blood, Tissues and Cells.^31^ This legislation mandates that SoHO entities should not offer financial rewards or other inducements to donors, a practice that commercial milk companies apply without either practical or emotional support for providers.^32 33^

Future funding for UK HMBs, particularly in England and Wales, therefore needs to be urgently considered especially since the recent British Association of Perinatal Medicine guidance and forthcoming WHO global minimum standards.^2 3^ Costs that would be obligatory in other SoHO provision,^34^ such as donor and recipient registries, coordinated tracking and tracing systems and an adverse event reporting and tracking system currently do not exist and are not factored into this costing analysis. DHM usage is predicted to increase by 25–50% in the UK^18^ but increased costs per litre may be proportionately less because of economies of scale with efficient use of staffing and equipment.

A notable limitation is it is difficult for all HMB leads to disentangle their varied workstreams and core costs to provide accurate costings. Additionally, not all HMBs provided costs; hence the costings presented here are likely an under-representation of the true costs for providing DHM to neonatal unit settings. Additionally, some key costs were not included in the analysis as they were either not relevant (eg, transportation, currently provided by blood bike volunteers or local volunteers) or do not currently take place (eg, adverse event reporting, accreditation, staff training). All HMBs have needed charitable funding for set-up costs, equipment purchases and for refurbishments. Future studies should assess the contributions from charities and how to ensure robust service continuity.

## Conclusion

This is the first survey to understand the costs of operating HMBs in a single country, the UK, highlighting large variations in costs and likely under-reporting of the true costs of a high quality, assured and sustainable service. This preliminary study should be repeated in a different timescale to support transparency in the determination of fair tariffs for HMBs and future decision-making on the development of nationally agreed cost recovery programmes for DHM. HMBs in other countries, including in low-income and middle-income settings, will be able to use the tools developed here. This data can be used to develop cost-saving innovations and for planning both clinical trials into DHM use and equitable milk banking services that ensure sustainable long-term service continuity.

## Data Availability

Data are available upon reasonable request.
